# Lysosome-Mitochondrial Crosstalk in Cellular Stress and Disease

**DOI:** 10.3390/antiox14020125

**Published:** 2025-01-22

**Authors:** Szilvia Kiraly, Jack Stanley, Emily R. Eden

**Affiliations:** UCL Institute of Ophthalmology, London EC1V 9EL, UK; szilvia.kiraly.17@ucl.ac.uk (S.K.); j.stanley.22@ucl.ac.uk (J.S.)

**Keywords:** lysosomes, mitochondria, crosstalk, membrane contact sites

## Abstract

The perception of lysosomes and mitochondria as entirely separate and independent entities that degrade material and produce ATP, respectively, has been challenged in recent years as not only more complex roles for both organelles, but also an unanticipated level of interdependence are being uncovered. Coupled lysosome and mitochondrial function and dysfunction involve complex crosstalk between the two organelles which goes beyond mitochondrial quality control and lysosome-mediated clearance of damaged mitochondria through mitophagy. Our understanding of crosstalk between these two essential metabolic organelles has been transformed by major advances in the field of membrane contact sites biology. We now know that membrane contact sites between lysosomes and mitochondria play central roles in inter-organelle communication. This importance of mitochondria–lysosome contacts (MLCs) in cellular homeostasis, evinced by the growing number of diseases that have been associated with their dysregulation, is starting to be appreciated. How MLCs are regulated and how their coordination with other pathways of lysosome–mitochondria crosstalk is achieved are the subjects of ongoing scrutiny, but this review explores the current understanding of the complex crosstalk governing the function of the two organelles and its impact on cellular stress and disease.

## 1. Introduction

Subcellular compartmentalization into membrane-bound organelles allows the separation of specialized cellular functions but also generates the need for intra-cellular communication. Recent years have witnessed substantial advances in our understanding of nonvesicular communication between organelles at highly regulated domains termed membrane contact sites (MCSs) [1]. Increasingly recognized as key regulators of diverse cellular processes, MCSs, where neighboring organelles are in very close apposition (~5–40 nm apart), provide platforms for protein interactions, signaling events, lipid exchange, and calcium flux [2].

**Figure 1:**
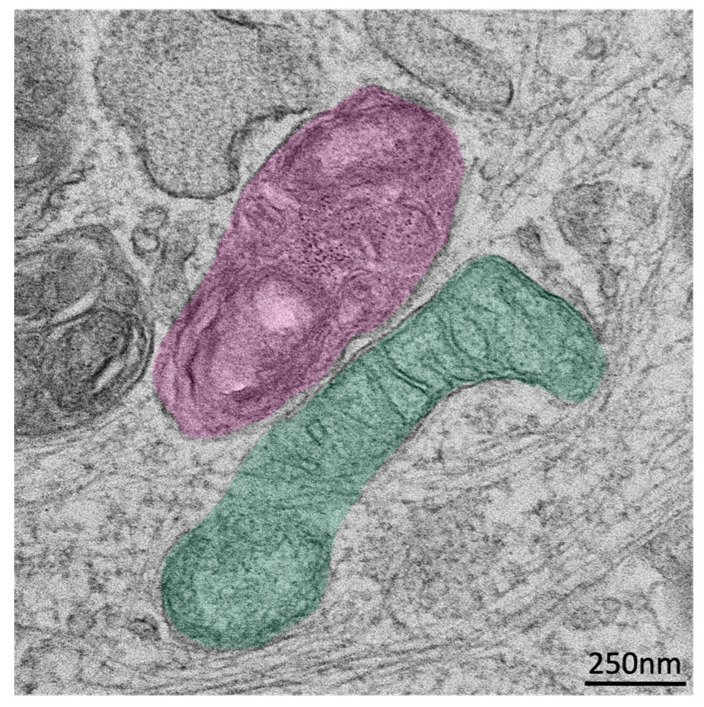
Electron micrograph of a mitochondria:lysosome contact (MLC). A lysosome (false-colored magenta) and mitochondria (false-colored green) are shown forming extended MLCs in fibroblasts from an NPC patient lacking functional NPC1. Scale bar, 250 nm.

Both mitochondria and lysosomes are essential for regulating cellular metabolism, and the dysfunction of both organelles is implicated in a variety of neurodegenerative diseases. In the lysosomal storage disorder, Niemann Pick type-C (NPC), for example, loss of function of lysosomal lipid transport proteins NPC1 or NPC2 causes the accumulation of lysosomal lipids, but, intriguingly, additionally results in mitochondrial dysfunction [3], indicating functional crosstalk between the two organelles. While signaling pathways contribute to this crosstalk (e.g., via mTORC1 hyperactivation in NPC [4]), the extensive and expanded contact between lysosomes and mitochondria in cellular models of NPC [5] also implicates MCSs in the mechanism of their coupled dysfunction (Figure 1). Since their identification in yeast approximately a decade ago, mitochondria:lysosome contacts (MLCs) have been implicated in regulating far-reaching processes important for the maintenance of cellular homeostasis. Here, we review the current understanding of the cellular mechanisms of crosstalk between lysosomes and mitochondria and their coupled dysfunction in neurodegenerative diseases.

Lysosomes: through the endocytic pathway, extracellular cargo is internalized into early endosomes (EEs), where it is sorted for recycling to the plasma membrane, for retrograde transport to the trans-Golgi network (TGN), or for degradation in the lysosome. Endosomes undergo a maturation process involving dynein-dependent traffic along microtubules toward the microtubule-organizing center, normally located toward the center of the cell near the nucleus. Maturation involves progressive luminal acidification as changes occur in the lipid and protein composition, notably including a switch in endosomal small GTPases, from Rab5 predominating on EE to Rab7 on late endosomes (LE) [6,7,8].

Lysosomes are the terminal organelles of the endocytic cycle that possess degradative capacity due to their low intra-lumenal pH and abundance of acid hydrolases. While these hydrolases are present in endosomes at earlier stages of maturation, they function more efficiently in an acidic environment. The acidic luminal pH is maintained by the large vacuolar channel, V-type ATPase (v-ATPase), which hydrolyzes ATP to pump protons into the lumen. V-ATPase assembly correlates with Rab7 activity, indicating that the Rab protein switch in the endosome maturation is key for the acidification of these organelles [9].

The abundance of hydrolases and low pH have given lysosomes the stereotype of the recycling and waste center of the cell. Lysosomes, in fact, have a much more diverse arsenal of roles, including calcium signaling and lipid homeostasis. The far-reaching roles of the lysosome become apparent in diseases termed lysosomal storage disorders (LSDs), where the consequences of impaired lysosomal trafficking and accumulation of poorly digested material are complex, often involving mitochondrial dysfunction. Continuing with NPC as an example, this rare, progressive neurodegenerative LSD is caused by a defect in lysosomal lipid egress that manifests with severe neurological symptoms, due to predominant neuronal vulnerability, but also affects visceral organs. NPC results from the loss of function of LE/lysosome lipid transport proteins NPC1 (95% of cases) or NPC2 (5% of cases), leading to the accumulation of unesterified cholesterol and glycosphingolipids within the LE/lysosomes, but the associated mitochondrial dysfunction is thought to be a key driver of disease pathology [10].

Mitochondria: thanks to their key role in energy metabolism and ATP production, mitochondria are known as the cell’s powerhouse. Beyond this, mitochondria are the major sites of various cellular processes such as calcium homeostasis, heme synthesis, apoptosis, and the iron–sulfur cluster biosynthetic pathway. Defects in mitochondrial function are associated with neurodegenerative diseases including Parkinson’s disease (PD), Alzheimer’s disease (AD), and dementia. Alterations in mitochondrial structure and function lead to increased reactive oxygen species (ROS) and decreased ATP production, contributing to neuronal damage [11,12,13,14]. Mitochondrial dysfunction often coincides with lysosomal dysfunction in neurodegenerative diseases and LSDs, including NPC, where mitochondrial dysfunction has been linked to altered calcium homeostasis, increased oxidative stress, and apoptosis. The abnormal mitochondrial morphology, alterations in autophagy, and deficient oxidative phosphorylation reported in NPC1-deficient cells [15,16,17,18] demonstrate the potential for defects of the lysosome to impact mitochondrial function. Similarly, the converse is also true: mitochondrial dysfunction can lead to alterations to lysosomal function. For example, depletion of AIFM1 (an apoptosis-inducing factor essential for respiratory chain function), OPA1 (necessary for mitochondrial fusion), or PINK1 (involved in respiratory chain quality control and mitophagy) in mouse embryonic fibroblasts leads to lysosomal dysfunction shown by the expansion of lysosomal (LAMP1-positive) vesicles, which become nonacidic and lose their hydrolytic activity [19].

## 2. Lysosome-Mitochondria Crosstalk and Lysosome pH

Since lysosomal function is central to cellular quality control and protein degradation, lysosomes play an immense role in maintaining cellular homeostasis by regulating cell signaling events, nutrient homeostasis, and removing cellular debris including pathogens and misfolded proteins using the autophagy–lysosome pathway [20,21].

Most lysosomal functions rely on maintaining optimal lysosomal lumen acidity (pH between 4.5 and 4.7), with protein degradation being achieved by acid hydrolases within the lumen [20]. Elevated lysosomal pH and the associated lack of a degradative capacity are thought to contribute to the pathogenesis of multiple neurodegenerative diseases [22,23]. The acidic environment of the lysosomal lumen is the consequence of an electrochemical gradient regulated by v-ATPase with the help of the chloride channel CLC7 [24]. As the pH increases, the activity of acid hydrolases and lipases decreases, and the lysosome becomes dysfunctional. The reduced degradative capacity of the lysosome has downstream effects on mitochondrial quality control (MQC), as mitophagy, the process by which damaged mitochondria are degraded, requires functional lysosomes for resolution. Accordingly, decreased acidification of the vacuole (yeast equivalent of the lysosome) has been shown to lead to impaired mitochondrial function [25]. It has also been shown that, in fibroblasts from Down syndrome patients, which are predisposed to early-onset Alzheimer’s disease, the extra chromosome 21 encoding the amyloid precursor protein (APP) causes increased levels of the β-cleaved carboxy-terminal fragment of APP which impairs lysosomal acidification and function through the inhibition of the v-ATPase as well as dysfunctional mitophagy [26].

Defects in the activity of the lysosomal enzyme glucocerebrosidase also impact mitochondrial function and dynamics, again highlighting the importance of crosstalk between the two organelles in health and disease. β-glucocerebrosidase (GBA1/GCase) catalyzes the conversion of glucosylceramide (GlcCer) into glucose and ceramide and its loss leads to Gaucher’s disease. Triacylglycerol and cholesterol have been found to be increased in Gaucher’s disease patient lymphoblasts and in RAW macrophages where GlcCer breakdown is inhibited by conduritol B-epoxide due to a rise in lysosomal pH [27]. Genome-wide association studies reveal that GBA1 gene mutations are a major risk factor in PD, and patients carrying GBA1 mutations have more severe cognitive symptoms [28,29]. Decreased GCase activity in both GBA1-Parkinson’s patient neurons and GCase inhibitor-treated cells is associated with extended MLCs, elevated lysosomal pH, and mitochondrial dysfunction [30]. Increased MLCs and mitochondrial dysfunction in these cells can be partially rescued by overexpressing TBC1D15 to facilitate contact site disassembly. Increased lysosomal pH has also been reported in NPC1 patient fibroblasts [31,32], and treatment with the nonlysosomal glucocerebrosidase (GBA2) inhibitor rescued the elevated endolysosomal pH and restored disturbed ceramide trafficking (measured by BODIPY-LacCer) [32]. Mechanistically, GlcCer has been proposed to bind and activate v-ATPase, the expression of which increases following GBA2 inhibition. More recently, an internal mitochondrial targeting sequence has been identified in GCase that promotes GCase import into the mitochondria, where it modulates mitochondrial complex I integrity, activity, and mitochondrial respiration in iPSC-derived neurons, perhaps suggesting an additional, more direct role of GCase in mitochondrial bioenergetics [33]. However, as far as we are aware, no studies have yet reported a direct correlation between elevated lysosomal pH and effects on mitochondrial function and mitochondrial ROS production in mammalian cells.

On the other hand, mitochondrial dysfunction has been shown to affect both lysosomal function and pH. Acute disruption of mitochondrial respiration in T cells, where the transcription factor A (TFAM) is lacking or pharmacologically inhibited, has been found to lead to reduced lysosomal Ca^2+^ mobilization, increased p62 and sphingomyelin, and reduced lysosomal degradative capacity. This, in turn, has been shown to trigger lysosomal transcription factor EB (TFEB)-dependent lysosomal biogenesis [34]. Chronic disruption of the mitochondrial respiratory chain has been shown to increase lysosomal pH, inhibit lysosomal proteolytic activity, and repress lysosomal Ca^2+^ channels due to decreased AMPK signaling [19,35,36]. Importantly, antioxidants can rescue lysosomal function, indicating that oxidative stress plays a crucial role in the lysosomal phenotype arising as a consequence of mitochondrial dysfunction [19]. Mitochondrial translation defects can also cause impaired lysosomal pH and inhibited lysosomal proteolytic activity due to decreased nicotinamide adenine dinucleotide (NAD+) [37]. P32-deficient mice have impaired mitochondrial ribosome formation, leading to loss of mitochondrial translation and function as well as decreased lysosomal acidification and NAD+ synthesis. Treatment of p32 knockout mouse embryonic fibroblasts with nicotinamide mononucleotide (NMN) or overexpression of the NAD+ synthesis enzyme Nmnat3 restored lysosomal acidification [37].

## 3. Lysosome-Mitochondria Crosstalk and Calcium and Iron Flux

Calcium: intra-cellular Ca^2+^ serves as an important second messenger for the control of a wide variety of cellular functions, and the multiple responses evoked by changes in cytosolic Ca^2+^ concentrations necessitate its tight regulation. While the ER is the main intra-cellular Ca^2+^ store, mitochondria and lysosomes can also act as calcium signaling hubs, and the importance of MCSs between these organelles in calcium homeostasis is becoming increasingly apparent, with Ca^2+^ shown to be transported across inter-organelle junctions at mitochondria:ER contacts (MERCs) [38,39], ER:lysosome contacts (ERLCs) [40,41], and MLCs [42].

Ca^2+^ transport at MERCs is mediated by a complex consisting of a quartet of proteins (summarized in Table 1): the ER-resident Ca^2+^ channel inositol 1,4,5-triphosphate receptor (IP3R) and the mitochondrial voltage-dependent anion-selective channel (VDAC1), both of which regulate Ca^2+^ transfer, and the glucose-regulated protein 75 (GRP75) and DJ-1, both of which bind both Ca^2+^ channels and act as adaptors to maintain the complex [43]. Ca^2+^ is released from the ER through IP3R in response to stimulation by multiple different factors, including cytosolic and ER Ca^2+^ concentration, ATP, protein interaction, and phosphorylation [44]. Ca^2+^ is imported into the mitochondria through VDAC1 on the outer mitochondrial membrane and into the matrix through the mitochondrial Ca^2+^ uniporter (MCU). Interestingly, this inter-organelle transfer has recently been shown to require the optimal distance of 20 nm between apposing membranes at MERCs [45].

Lysosomes also serve as a major store of cellular Ca^2+^, which is maintained at approximately 0.5 mM, a similar concentration to that of the ER [65]. Like the ER, acidic organelle Ca^2+^ stores can also be imported by mitochondria at MCSs. Work by Peng et al. [42] demonstrated Ca^2+^ release from lysosomes via the transient receptor potential (TRP) mucolipin 1 (TRPML1) channel. The mechanism of mitochondrial import is analogous to that at MERCs (summarized in Table 1): released Ca^2+^ is taken up into mitochondria through the VDAC1 channel on the outer mitochondrial membrane (OMM) before transport through the MCU in a contact-site-dependent and ER-independent manner [42]. Lysosomal Ca^2+^ is depleted in NPC1-deficient cells, potentially due to an accumulation of sphingosine [66] which may act as an agonist of the TRPML1 channel or two-pore channel (TPC)-1, promoting increased Ca^2+^ release from lysosomes [67]. No change in lysosomal pH has been detected following NPC1 inhibition, but lysosomal Ca^2+^ has been found to decrease [66]. In contrast, a different study found that there are comparable Ca^2+^ levels in NPC1-deficient cells compared to controls but a reduced release from lysosomes due to TRPML1 inhibition by sphingomyelin which accumulates in NPC [68]. Perhaps counter-intuitively, given the reduced release from lysosomes, mitochondrial Ca^2+^ has been reported to be increased in NPC, contributing to mitochondrial dysfunction [69]. The voltage-gated potassium channel K_V_2.1 tethers ER:plasma membrane contacts through phosphorylation-dependent interactions with ER-localized VAPs. Through interactions with K_V_2.1, voltage-gated L-type Ca^2+^ channels (Ca_V_1) become clustered at the contact site, promoting Ca^2+^ entry. K_V_2.1 phosphorylation by cyclin-dependent kinase 5 (CDK5) regulates its interaction with VAP and, therefore, the clustering of Ca^2+^ channels at the contact site. Hyperactivation of mTORC1 in NPC is thought to underly channel clustering through AMPK inhibition and the consequent increased CDK5 activity. Channel clustering increases SERCA-dependent Ca^2+^ entry into the ER, followed by a rapid release via IP_3_ receptors at MERCs to promote a neurotoxic rise in mitochondrial Ca^2+^.

Mutations in MCOLN1, which codes for the TRPML1 channel, are associated with mucolipidosis type IV (MLIV), a disease resulting in impaired development, vision, and motor function. Fibroblasts from MLIV patients with TRPML1 mutations have been found to have increased and elongated mitochondria–lysosome contacts and lysosome and mitochondrial dysfunction [42]. Moreover, depletion of TRPML1 also causes an increase in both MLCs and mitochondrial Ca^2+^, thought to be due to Ca^2+^ transfer from other lysosomal Ca^2+^ channels at the expanded interface between organelles [70]. This Ca^2+^ homeostasis may also be important in lysosomal repair, as lysosomal Ca^2+^ levels are essential for ESCRT-mediated repair and may be concentrated at MLCs established by the interaction between HKDC1 and VDAC1 (Table 1, [71]). The mitophagy proteins PINK1 and parkin have additionally been implicated in Ca^2+^ homeostasis, with the suggestion that PINK1 deficiency can lead to an overload of mitochondrial Ca^2+^ and an increase in ROS production [72,73]. This, potentially, implicates PINK1 in the regulation of MLCs, just as it plays a role in MERCs: PINK1 depletion in M17 dopaminergic cells reduces the number of MERCs and increases the distance between neighboring ER and mitochondria [74].

Iron: like Ca^2+^, iron can be stored in both lysosomes and mitochondria and is required for many cellular processes, including, but not limited to, DNA synthesis, mitochondrial homeostasis, and cell proliferation. Mitochondria are the major cellular sites of iron utilization. Mitochondrial iron supports the biosynthesis of heme and iron–sulphur (Fe–S) clusters, which act as cofactors of enzymes in the tricarboxylic acid (TCA) cycle and the respiratory chain complexes, as well as of many cytosolic enzymes. To mitigate oxidative stress caused by their high iron levels, mitochondria utilize an iron storage protein known as mitochondrial ferritin (FtMt). This protein shares structural and functional similarities with cytosolic ferritin. FtMt plays a vital role in mediating lysosomal degradation of mitochondria by mitophagy triggered by low iron levels [75].

Lysosomes are gaining recognition for their role in the regulation of iron-related metabolic pathways. However, our knowledge of the intra-cellular mechanisms that connect lysosomes and iron metabolism, and how this connection regulates essential cellular processes, remains limited. Iron is taken up by mitochondria via mitoferrin-1 and -2 solute carriers on the inner mitochondrial membrane [76] prior to incorporation into matrix Fe-S clusters, which act as cofactors for various enzymes in the citric acid cycle and electron transport chain [77]. While the mechanism of iron delivery between lysosomes and mitochondria has not been fully elucidated, current models include chaperone-guided cytosolic transit and direct inter-organellar transfer through transient interaction (“kiss and run”) [78]. Iron bound to transferrin (Tf) is taken up by the cell and released within the endosome upon acidification. MLC formation involving VDAC1 or the divalent metal transporter-1 (DMT1) on the outer mitochondrial membrane could provide a physical tether to allow iron transfer [79,80]. A recent preprint provides further mechanistic insights. In melanoma cell lines, a short-chain dehydrogenase/reductase family member, 3-hydroxybutyrate dehydrogenase 2 (BDH2) at MLCs, was found to generate 2,5-dihydroxybenzoic acid (2,5-DHBA), which shuttles iron from lysosomes to mitochondria to support oxidative phosphorylation (OXPHOS) and ATP production, which, in turn, is utilized by lysosomes to maintain a low pH through v-ATPase activity [79]. The inter-organelle transfer of iron is important in the progression of melanoma in the transition into ‘invasive’ mesenchymal-like cells which has been suggested to promote metastasis. The mesenchymal-like cells have been shown to have an increase in lysosomal iron accumulation, reduced mitochondrial iron, and reduced ATP production. This is accompanied by reduced levels of BDH2 and an associated increased sensitivity to the programmed cell death pathway ferroptosis, with BDH2 overexpression in the mesenchymal-like cells being sufficient to prevent ferroptosis [81].

Ferroptosis is triggered by an accumulation of intra-cellular iron leading to lipid peroxidation and membrane damage. The depletion of mitochondrial GSH and glutathione peroxidase 4 (GPX4) leads to the accumulation of ROS and to cell death [82]. Additionally, as a store of iron and the location of ROS production, lysosomes play an important role in ferroptosis. Suppression of lysosomal ROS production by lysosome inhibitors, such as bafilomycin, reduces cells’ sensitivity to ferroptosis [83]. Lysosomal degradation of extracellular proteins can also protect from ferroptosis. Depletion of extracellular cystine can trigger ferroptosis, and this can be rescued by either mTOR inhibition or by lysosomal recycling of ingested albumin to stabilize GSH levels and prevent lipid peroxidation [84].

## 4. Lysosome–Mitochondria Crosstalk and Lipid Homeostasis

Cholesterol: cholesterol is a critical component of all animal cell membranes and significantly affects membrane fluidity, permeability, curvature, and membrane–protein interactions [85]. Mitochondria acquire cholesterol through several pathways from different cellular pools [86]. Mitochondrial cholesterol accounts for only 2–4% of total cellular cholesterol and is maintained within a narrow range to regulate steroid and oxysterol synthesis and to ensure mitochondrial function [87]. Mitochondria can synthesize oxysterols from cholesterol and increased mitochondrial cholesterol could lead to elevated oxysterols in steroidogenic cells. Cholesterol is transported from the OMM to the inner mitochondrial membrane (IMM) by the steroidogenic acute regulatory protein 1 (STARD1, StAR) [87,88]. STARD1 is part of the STAR family that consists of lipid transport protein with steroidogenic acute regulatory protein-related lipid transfer (START) domain that moves various lipids such as cholesterol, oxysterols, and sphingolipids [89,90]. Mice with STARD1 deletion develop congenital lipoid hyperplasia and die within 7–10 days after birth, suggesting that no other STAR family protein can compensate for the loss of STARD1 [91]. Interestingly, a recent study has demonstrated an inverse relationship between the lysosomal acid ceramidase and STARD1 expression, dependent on lysosomal cholesterol [92]. Liver and brain from NPC1 knockout mice and NPC patient fibroblasts have reduced acid ceramidase and an increased expression of STARD1. Overexpression of acid ceramidase or depletion of cellular cholesterol correct the STARD1 expression levels and increase mitochondrial function.

Increased cholesterol transport to mitochondria has been demonstrated in NPC1-deficient cells [93], a process that is dependent on the START domain-containing protein STARD3 (also known as MLN64), although it remains to be seen whether this transfer happens directly at MLCs [15,86]. STARD3 contains an MLN64 N-terminal (MENTAL) domain that anchors the protein to the LE/lysosome membrane and a C-terminal START domain that can transfer cholesterol [63,64,94,95]. STARD3 additionally contains a central FFAT motif (two phenylalanines in an acidic tract) that binds ER-localized VAPs and motile sperm domain-containing 2 (MOSPD2) to tether ER:LE/lysosome MCSs. Like the K_V_2.1 channel, the interaction with VAP is dependent on the phosphorylation of a key serine residue in STARD3’s FFAT motif (S209), but, in the case of STARD3, the interaction with VAP proteins builds a molecular machine able to transfer cholesterol that has been shown to mediate the transport of newly synthesized cholesterol from the ER to endosomes [59,96,97]. The hypothesis that, in cholesterol storage disease conditions, STARD3 may also function to facilitate lysosome to mitochondria cholesterol transport is supported by data from NPC1 patients showing increased circulating oxysterols which are improved by recovery of mitochondrial glutathione (mGSH) levels [98,99]. Balboa et al. (2017) also demonstrated that STARD3 overexpression leads to mitochondrial alterations, including decreased mitochondrial membrane potential (MMP) as well as increased superoxide production, suggesting that STARD3 overexpression leads to mitochondrial dysfunction by increasing mitochondrial cholesterol levels [100].

**Figure 2:**
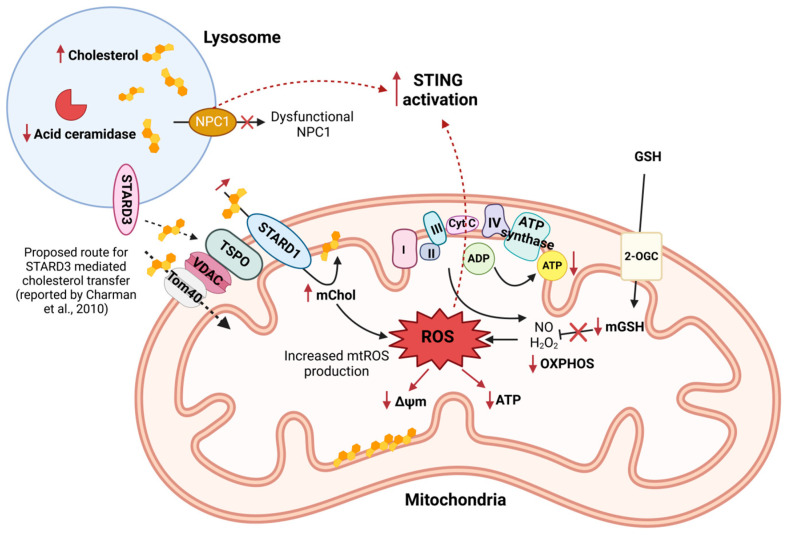
Coupled dysfunction of lysosomes and mitochondria in Niemann–Pick type C (NPC) disease, leading to increased ROS production and innate inflammation. Illustration of crosstalk between lysosomal and mitochondrial dysfunction in NPC. A defective NPC1 resulting in the accumulation of lysosomal cholesterol and sphingolipids is also associated with reduced acid ceramidase activity and increased expression of the mitochondrial cholesterol import protein STARD1. STARD3 on LE/lysosomes and mitochondrial Tom40, TSPO, and StARD1 have been implicated in mitochondrial cholesterol (mChol) accumulation [15]. Increased mChol disrupts membrane fluidity and impairs electron transport chain (ETC) complexes, leading to elevated mitochondrial reactive oxygen species (mtROS) production and reduced oxidative phosphorylation (OXPHOS). Consequences include decreased mitochondrial membrane potential (Δψm) and ATP synthesis. In parallel, mitochondrial glutathione (mGSH) depletion through reduced 2-oxoglutarate carrier (2-OGC) activity due to a change in membrane fluidity exacerbates oxidative damage [99,108,109]. Increased mtROS activates innate inflammatory pathways including cGAS/STING signaling, and dysfunctional NPC1 is unable to recruit STING to the lysosome for degradation, further increasing the inflammatory response. This cross-organelle dysfunction highlights a critical axis driving cellular damage and inflammation in NPC pathology. The solid arrows represent direct transport or transfer processes, while the dashed arrows indicate proposed effects or signaling pathways, such as the proposed route of STARD3 mediated cholesterol transfer [15]. The red arrows highlight pathological outcomes in NPC, such as decreased mitochondrial membrane potential (Δψm) and the crossed-out arrow represents inhibited reduction of mitochondrial reactive oxygen pieces due to decrease in mGSH transport. Image created with BioRender.com (accessed on 24 November 2024).

Another potential mechanism for the connection between cholesterol distribution and mitochondrial dysfunction is mTORC1 signaling, which is perturbed under conditions of high lysosomal cholesterol and is subject to regulation by lysosomal GTPases, such as Ras-associated protein 7 (Rab7) in response to nutrients and stress [101,102,103]. Of note, it has been shown that overexpressing annexin-A6, a Ca^2+^-dependent membrane-binding protein, induces an NPC-like phenotype and promotes Rab7 inactivation via TBC1D15-mediated hydrolysis [104,105,106,107]. Consequently, the same study found that silencing annexin-A6, with the associated loss of TBC1D15 recruitment and an increase in GTP-bound Rab7, alleviates cholesterol accumulation in NPC1 mutant cells in a STARD3-dependent manner. As illustrated in Figure 2, the accumulation of cholesterol in mitochondrial membranes in NPC1-deficient cells has been reported to impair the transport of mGSH, which is dependent on the inner membrane fluidity, and decreased mGSH levels have been found in the brain and liver of NPC mice [99,108].

GSH is an important antioxidant containing a redox-active thiol group which oxidizes when target molecules are reduced by GSH [109]. Mitochondrial GSH is crucial in balancing mitoROS through the metabolism of hydrogen peroxide. Furthermore, mGSH defends the mitochondrial membranes from oxidative damage by reducing the hydroperoxide groups on phospholipids. Therefore, in cells that are major sources of mitoROS such as hepatocytes, a decrease in mGSH levels can sensitize cells to cell death by ROS. This could, potentially, contribute to liver disease that is diagnosed in a significant amount of NPC patients. NPC patients are mostly diagnosed with hepatosplenomegaly, but many NPC patients suffer from elongated neonatal jaundice and liver failure. NPC is the second most common cause of neonatal cholestasis, and liver failure is the cause of death in 10% of patients [110,111].

Another potential consequence of increased oxidants and ROS by mitochondrial cholesterol loading is the effect on mitochondrial phospholipid composition. One of the most sensitive lipid species is cardiolipin, which is an anionic phospholipid residing only in the IMM close to the OXPHOS protein complexes and is essential for IMM structure integrity and for respiratory chain function [112,113,114]. Oxidative alterations of cardiolipin affect the activity of the respiratory chain leading to mitochondrial dysfunction and impact apoptosis by regulating the release of cytochrome c and the binding of the Bcl-2 family protein Bid to the outer mitochondrial membrane [115].

Phospholipids: other phospholipids, in addition to cardiolipin, play important roles in mitochondrial function, and there appears to be a level of reciprocal regulation of inter-organelle crosstalk and phospholipid homeostasis. Dysregulation of the phospholipid constituent ceramide caused by loss of GBA1/GCase is associated with elongated MLCs in neurons [30]. In yeast, contact sites between mitochondria and the vacuole, known as vacuole and mitochondria patch (vCLAMP), have been shown to regulate phospholipid transport between mitochondria and vacuoles [51]. Deletions of ER–mitochondria encounter structure (ERMES), together with vCLAMP, results in severe alterations to mitochondrial phospholipid composition such as accumulation of phosphatidylserine and decreased phosphatidylcholine. The yeast vCLAMP is linked via two distinct pathways: one involves mitochondrial Tom40 binding to the vacuolar sorting and fusion protein VPS39, which interacts with the vacuolar membrane via the Rab GTPase Ypt7, the other involves mitochondrial MCP1 binding to Vps13, which associates with the vacuolar membrane through interaction with Ypt35 [51].

Oxysterol binding protein (OSBP) family-related protein (ORP) ORP1L has recently been shown to mediate the transport of phosphatidylinositol 4-phosphate (PI(4)P) from lysosomes to mitochondria at three-way contact sites among LE/lysosomes, ER, and mitochondria [116], likely contributing to the role of MLCs in mitochondrial fission, discussed in more detail below. Whereas ORP1L transports cholesterol at LE/lysosome contact sites with the ER, it functions as MLCs at sites of mitochondrial division to transfer PI4P generated by lysosomal phosphatidylinositol-4 kinase activity to mitochondrial membranes, with ORP1L-depletion inducing mitochondrial elongation. PI4P also plays an important role in membrane repair mechanisms following lysosomal damage. Mass spectrometry and subsequent immunoblotting have identified enrichment of a phosphatidylinositol-4 kinase (PI4K2A) and members of the OSBP family and ORP proteins, such as ORP9 and ORP1L [117], in lysosomal membranes following lysosome membrane damage by L-leucyl-L-leucine methyl ester (LLOME) [118]. The consequent generation of PI(4)P results in the recruitment of multiple ORPs to damaged lysosomes, including the FFAT motif-containing ORP9, which tethers ER contact through interactions with VAPs [118]. PI(4)P generated from the lysosomes and transported to the ER is hydrolyzed by its ER-resident phosphatase, Sac1, generating a concentration gradient along which PI(4)P is transported, driving a lipid exchange mechanism that involves the ORP9/ORP11-mediated transfer of cholesterol and phosphatidylserine (PS) from the ER to the lysosomes [118]. Interestingly, triggering the release of lysosomal Ca^2+^ through TRPML1 by treatment with ML-SA1 leads to the rapid recruitment of PI4K2A to lysosomes, independent of lysosomal damage by LLOME [118], suggesting a role of Ca^2+^ signaling as a trigger for PI(4)P-mediated lipid exchange at ER:lysosome contacts in response to lysosome damage. The finding that NAADP-mediated Ca^2+^ release through the lysosomal two-pore channels (TPCs) promotes ER–endosome contact [41] is consistent with a key role of lysosomal Ca^2+^ release in regulating ER–lysosome contact. Lysosome damage causes a subsequent leakage of lysosomal proteases, which have been reported to decrease the levels of OMM proteins and lead to remodeling of the mitochondrial proteome to reduce the levels of IMM electron transport chain proteins in macrophage models [119].

## 5. Lysosome: Mitochondria Crosstalk and Mitochondrial Dynamics and Quality Control

**Figure 3:**
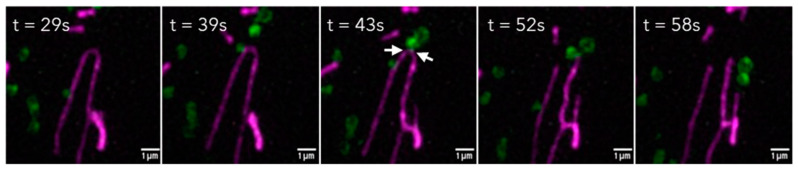
Lattice structured illumination microscopy data showing mitochondrial fission sites contacted by LE/lys in ARPE19. Live cell images generated using the Zeiss Lattice SIM 3 in ARPE19, with the mitochondrial marker PKmito ORANGE (magenta) and cells transiently transfected with the LE/lysosomal marker STARD3-GFP (green). Time-stamped images of the contact formation, mitochondrial fission, and subsequent untethering of the membrane contact site between mitochondria and LE/lysosomes. White arrows represent MCSs.

Mitochondrial fission*:* MLCs play a major role in maintaining mitochondrial dynamics by regulating fission (e.g., Figure 3). While examining MLCs, Wong et al. demonstrated dynamic MLC formation in HeLa cells and showed that these MLCs do not support bulk transfer of organelle content, are distinct from mitochondrial-derived vesicles (MDVs), and do not lead to mitophagy [47]. They, instead, identified LAMP1-positive LE/lysosome contact with mitochondria at 81.5% of the mitochondrial fission sites mediated by Rab7 on LE/lysosomes and the accumulation of the mitochondrial fission protein Fis1 on the outer mitochondrial membrane upon the initiation of fission [47]. Fis1 accumulation recruits Rab7-GTP activating protein (GAP) TBC1D15, which drives the hydrolysis of GTP that, in turn, inactivates Rab7, as it can no longer engage with its effectors and loses lysosomal localization, triggering MLC untethering [47]. Constitutively active mutants of Rab7, such as Q67L, and GAP-inactive mutants of TBC1D15 have been shown to decrease the rate of fission and increase the minimum duration of MLC, without increasing the percentage of lysosomes forming mitochondrial contacts, indicating that the Rab7 GTP hydrolysis by TBC1D15 can contribute to MLC disassembly [47]. VPS13A, which colocalizes largely to mitochondria, interacts with Rab7A and may stabilize MLCs [120]. Drp1, a mitochondrial dynamin family GTPase, has been shown to interact with Rab7 when phosphorylated on Ser616 [121]; a summary of these interactions at MLCs is provided in Table 1. The dephosphorylation of Drp1 Ser616 is mediated by protein phosphatase 2A B56 gamma subunit (B56γ), and the knockdown of B56γ results in increased mitochondrial fission. Interestingly, mice that were implanted with B56γ-overexpressing cells showed slower tumor growth and an increased expression of apoptosis-related proteins, increasing hepatocellular carcinoma cells chemosensitivity, suggesting that reducing mitochondria–lysosome crosstalk mediated by the Rab7-Drp1 interaction could be a potential therapeutic target for cancer treatment [121].

Perhaps unsurprisingly, given their role in mitochondrial dynamics, perturbed MLCs have been implicated in disease, as summarized in Table 2. *GBA1* mutations have been implicated in PD pathology, with patients suffering more severe cognitive dysfunctions [122]. *GBA1* mutant neurons from Parkinson’s disease (PD) patients have been shown to have significantly decreased TBC1D15 levels and stabilized MLCs, correlating with the loss of activity of the lysosomal GCase (encoded by *GBA*) [30,122]. Expressing TBC1D15 to promote Rab7 hydrolysis and MLC disassembly in GBA1 mutant PD patient cells leads to a reversal of the mitochondrial dysfunction phenotype, increasing ATP production [30].

Rab7-dependent MLCs have also been implicated in Charcot–Marie–Tooth type 2B disease, an axonal sensorimotor neuropathy. The Rab7 V162M GTPase mutation has been shown to significantly increase MLC contact duration in peripheral sensory neurons, and overexpression of TBC1D15 in these mutant neurons has been shown to restore the contact duration to control levels [130]. The consequences of increased contact duration due to the Rab7 mutant were increased lysosomal size, increased mitochondrial density, and decreased motility in neurons, which, again, could be rescued by TBC1D15 [130].

As discussed above (phospholipids), mitochondrial fission events are influenced by the ER as well as the LE/lysosomes at three-way contact sites [131]. Lysosomes are present at approximately 60% of the sites of mitochondrial division, of which 91% is involved prior to ER recruitment [116]. ORP1L has been identified at 58.1% of the division events in a separate experiment, indicating its recruitment to MLCs during mitochondrial division.

Mitophagy: mitophagy is the term used to describe the macroautophagy of damaged mitochondria, ultimately culminating in their fusion with lysosomes. Under conditions of mitochondrial membrane depolarization, mutagenic stress, or proteotoxicity, mitophagy is mediated by the PINK1/parkin pathway. PTEN-induced serine/threonine kinase 1 (PINK1) is a mitochondrial protein that is imported into the mitochondria where, under normal physiological conditions, it is cleaved by proteases such as PARL between amino acids Ala103 and Phe104 [132,133]. This produces a 52 kDa form of PINK1 with an N-terminal Phe104 which is degraded by the ubiquitin–proteasome system (UPS) by the N-end rule [134]. Upon depolarization of the mitochondrial membrane, PINK1 is stabilized on the OMM, where it phosphorylates substrates and recruits the E3 ubiquitin ligase parkin. Upon phosphorylation of Ser65 by PINK1, parkin undergoes a change in conformation from a ‘closed’, autoinhibited state [135,136] to an active state where the cleft in which Ser65 lies becomes open, with the change in the structure causing the interaction between its ubiquitin-like domain and the RING1 domain to be broken [135]. Parkin substrates include Mfn2, VDAC1, and Miro [137,138]. It has been established that mitophagy is not the driver of MLC formation, since MLCs form in the absence of the autophagy machinery p62, Nuclear dot protein 52 (NDP52), optineurin (OPTN), neighbour of Brca1 gene (NBR1), and Tax1 binding protein 1 (TAX1BP1) [139], but contact sites with both ER and lysosomes may influence mitophagy. Mfn2 is a known tether of MERCs, and VDAC1 is present at both MERCs and MLCs, perhaps suggesting that mitochondria may need to disassociate from other organelles during the mitophagy process. Drp1, a key regulator of mitochondrial fission that is implicated in MLCs through interaction with Rab7, has been reported to be involved in the regulation of the mitophagic flux [116], again suggesting a link between MLCs and mitophagy. This shared machinery suggests that, although mitophagy is not required for MLCs, the two mechanisms may be related, with MLCs possibly playing a role in the regulation of mitophagy.

Recruitment and activation of parkin by PINK1 generates phosphorylated poly-ubiquitin chains resulting in the recruitment of ubiquitin-binding domain-containing autophagy receptors such as p62, OPTN, and NDP52 [140]. These autophagy receptors contain LC3-interacting motifs, enabling the recruitment of LC3-positive phagophores and autophagosome formation around the damaged and ubiquitin-labeled mitochondria. Following engulfment, the mitochondria-containing autophagosome undergoes fusion with a lysosome, providing an acidic environment in which the mitochondrial components can be degraded and recycled.

Mutations in PINK1 or parkin are both strongly associated with PD, indicating that defective mitophagy is a key driver in the pathogenesis of PD. PD is characterized by the loss of midbrain dopaminergic (DA) neurons in the substantia nigra par compacta (SNc) and by the presence of fibrillar aggregates called Lewy bodies [140]. Neurons are highly oxidative, relying heavily on the generation of ATP by OXPHOS to the point where the brain uses between 20 and 25% of the body’s oxygen intake despite only being approximately 2% of the total body volume [141,142]. DA neurons in the SNc have higher basal levels of mitochondrial OXPHOS than in other areas of the brain and operate much closer to maximum capacity at the basal state [143]. Neurons in the SNc are autonomously active, meaning they are constantly generating action potentials even in the absence of a conventional input [144]. As a result of their constant activity, they also demonstrate a higher rate of ROS production, higher mitochondrial density, and higher axonal branching [143]. Increased generation of ROS in mitochondria can damage integral mitochondrial proteins, resulting in impaired mitochondrial function that can lead to neuronal cell death. Consistent with ROS-induced mitochondrial damage, mitochondrial-specific ROS also trigger parkin-mediated mitophagy, while a reduction in ROS through the overexpression of the antioxidant superoxide dismutase-2, prevents mitophagy induction [145].

Vesicular communication between lysosomes and mitochondria can also occur through a form of microautophagy/mitophagy in which select regions of inner mitochondrial membranes form vesicles, thought to contain oxidized lipid species due to oxidative stress, which are degraded via the lysosome [146]. Together with mitophagy and the potential influence of MLCs over mitophagy, this highlights the complex crosstalk between these organelles in maintaining their respective homeostatic processes.

Transmitophagy and subsequent intercellular mitochondrial transfer are mechanisms that are exploited in a novel therapeutic method, mitochondrial transplantation. Donor cells with functional mitochondria are transplanted into tissues with suspected mitochondrial defects and can transfer healthy mitochondria to recipient cells via extracellular microvesicles, through tunnelling nanotubules (TNTs) or the release of free mitochondria which are taken up in a heparin–sulphate-dependent manner. Lysosome–mitochondria crosstalk may play a role in the release of mitochondria in extracellular microvesicles, with the release process being regulated by the Rab7 activation status [147]. Focusing on transfer via TNTs, OXPHOS-generated ROS are a key factor in mediating the growth of the TNT and in the direction of mitochondrial transport. The generation of ROS is increased in the cells undergoing stress or with mitochondrial damage and the elevated ROS levels activate p53 and the downstream Akt/PI3K/mTOR pathway to increase actin polymerization and formation of the TNT structure toward a donor cell [148]. During ROS-induced mitophagy [145], mitochondrial-derived damage-associated molecular patterns (mtDAMPs) are released which can promote mitochondrial biogenesis due to increased heme in the cells following the uptake of mtDAMPs. Donor mitochondria are transported in a Miro1-dependent [149] and MERC-dependent [150] manner toward the recipient cell where healthy mitochondria can rescue ATP production.

Apoptosis: apoptosis is also termed programmed cell death and there are two main accepted pathways that mediate apoptosis [151]. Exogenous apoptosis involves cell surface death receptors, while intrinsic apoptosis relies on mitochondrial involvement and lysosome–mitochondria crosstalk [152]. Mitochondria-dependent apoptosis is initiated under exposure to internal stimuli, including growth factor deprivation, hypoxia, DNA damage, oxidative stress, and Ca^2+^ overload, all of which are often linked to lysosomal damage.

Lysosome-dependent cell death (LDCD) is critically driven by lysosomal membrane permeabilization (LMP), which enables the release of lysosomal enzymes, such as cathepsins, into the cytosol [153]. Once released, cathepsins can trigger mitochondrial dysfunction and apoptosis, highlighting a key intersection of lysosomal and mitochondrial pathways in cell death regulation [154]. Cathepsins cleave the BH3-interacting domain death agonist (BID) into truncated BID (tBID), which promotes the oligomerization of pro-apoptotic Bcl-2 family member BAX [155]. The resulting BAX oligomers translocate to the mitochondrial outer membrane (OMM), facilitating the excessive formation of the mitochondrial permeability transition pore (mPTP) [156]. This leads to the release of cytochrome c into the cytoplasm, the activation of apoptosome formation, and the execution of mitochondrial-dependent apoptosis. Notably, cathepsin-mediated degradation of anti-apoptotic Bcl-2 proteins further enhances BAX activation, amplifying the apoptotic cascade. Notably, Bcl-2 family proteins like BNIP3 exemplify this interdependence; BNIP3 can localize to the mitochondria, facilitating its selective targeting for autophagic degradation via lysosomes [157]. This BNIP3-mediated mitophagy not only ensures mitochondrial quality control but also modulates ROS levels. LDCD is linked to ROS production, as lysosomal permeabilization and the subsequent release of cathepsins can increase mitochondrial ROS levels, exacerbating oxidative damage and cell death progression. Conversely, elevated ROS production in mitochondria induces lysosomal damage, highlighting a bidirectional interplay between these organelles in maintaining cellular homeostasis.

Mitochondrial Ca^2+^ signaling also plays an important role in apoptosis. Mitochondrial Ca^2+^ overload can stimulate the formation of the mPTP, through which Ca^2+^ and pro-apoptotic proteins such as cytochrome c are released [158]. ROS can exacerbate this process due to the peroxidation of cardiolipin which allows for the dissociation of cytochrome c and its release from mitochondria, through membranes via the mPTP or membranes with increased permeability, another effect of increased ROS [158]. In addition, caspase activity during the intrinsic apoptotic pathway, specifically caspase-9 activation, has been shown to be Ca^2+^-dependent [159].

## 6. Signaling Pathways in Mitochondria–Lysosome Crosstalk

AMPK and mTORC1: AMPK (AMP-dependent protein kinase) is a key sensor of cellular energy homeostasis and stress, and functions by activating various catabolic pathways and inhibiting anabolism [160]. AMPK signaling promotes TFEB-mediated lysosome biogenesis and assembly and the activation of v-ATPase and PIKfyve, a lysosomal membrane enzyme generating phosphatidylinositol 3,5-bisphosphate (PI(3,5)P2) [36,161]. Reduced PI(3,5)P2 in AMPK downregulation leads to decreased levels of the lysosomal Ca^2+^ channel TRPML1, with downstream effects on both autophagy and lysosomal biogenesis [162]. AMPK signaling likely plays an important role in inter-organelle crosstalk and is implicated in lysosomal dysfunction under conditions of chronic respiratory chain deficiency [35]. In this study, depletion of a respiratory chain complex III subunit (a model for chronic mitochondrial respiratory chain deficiency) was associated with reduced expression of TRPML1 and lysosomal impairment linked to the downregulation of AMPK signaling by induction of AMPK inhibitory tumor suppressor FLCN. Furthermore, AMPK promotes mitochondrial stress-activated autophagosome formation and autophagy [163]. Increased ROS generation by damaged mitochondria activates the MiT-TFE transcription factor TFEB, a master regulator of lysosomal biogenesis [164]. ROS can cause nuclear TFEB translocation by direct cysteine oxidation of TFEB or by activating TRPML1 [70,123,165,166]. This releases Ca^2+^ to the cytoplasm that activates Ca^2+^-dependent phosphatase calcineurin and calcineurin-dependent TFEB dephosphorylation and activation [162,166,167]. This allows the cell to increase its capacity for mitophagy and shows that TRPML1 can act as a ROS sensor, mitigating oxidative stress and autophagy [166]. Nuclear TFEB translocation induces peroxisome proliferator-activated receptor gamma coactivator 1-alpha (PGC1α), which induces the activity of several transcription factors that are involved in mitochondrial biogenesis, glucose homeostasis, and lipid oxidation [21]. On the other hand, in skeletal muscles, TFEB has been shown to be the central coordinator of mitochondrial function that activates the expression of several mitochondrial biogenesis genes, including TFAM, and increases the expression of mitochondrial enzymes [168].

It should be noted that, while other research has shown that ROS can also activate AMPK signaling, it has not been shown that the activation of TRPML1 by ROS depends on AMPK [169]. It is, however, clear that AMPK is a key regulator of mitochondrial and lysosomal stress responses and autophagy and is an important player in functional crosstalk between the two organelles.

mTORC1 works as a key counterbalance to AMPK and regulates anabolic pathways. Mitochondrial or lysosomal disease models are often described with AMPK downregulation and mTORC1 hyperactivation (reviewed in [170]). mTORC1 monitors the synthesis of fatty acids and sterols by increasing the expression and proteolytic processing of the master lipogenic transcription factors, SREBP1c and SREBP2, thereby promoting cell proliferation [171,172]. mTORC1 mediates mitochondrial biogenesis, and inhibition of mTORC1 causes reduced mitochondrial function [173]. However, under conditions of mTORC1 hyperactivation, inhibitory molecules such as rapamycin can help cells reserve ATP by reducing high-ATP-required functions, including protein translation and mitochondrial biogenesis that are often upregulated in chronic mitochondrial stress [174].

Cholesterol is a key activator of the mTORC1 kinase, increasing mTORC1 recruitment to the lysosomal membrane through the cholesterol sensor Rag guanosine triphosphatases (GTPases), establishing lysosomes as a central hub for growth regulation [175]. In the case of NPC where the cholesterol transporter NPC1 is dysfunctional, lysosomal cholesterol accumulation stimulates the Rag-dependent activation of mTORC1. NPC1 can bind to SLC38A9, a lysosomal transmembrane protein that inhibits mTORC1 signaling [176,177,178] and contains a cholesterol recognition amino acid consensus (CRAC) motif in its transmembrane domain as well as an inverted recognition motif called CARC that work together in sensing cholesterol [179]. SLC38A9 is required for mTORC1 recruitment to the lysosomes in response to LDL, and reintroducing the wild-type SLC38A9 protein to SLC38A9-depleted cells rescue mTORC1 activity while CRAC and CARC mutants fail, showing that the mTORC1 scaffolding complex relies on SLC38A9 to sense cholesterol [176]. Whereas SLC38A9/cholesterol promote mTORC1 activation, NPC1 acts as a negative regulator of mTORC1 and associates with the mTORC1 scaffolding complex to signal cholesterol depletion. mTORC1 hyperactivation is also thought to contribute to mitochondrial dysfunction in NPC which is improved by mTORC1 inhibition, but without reversal of lysosome cholesterol accumulation [4].

STING pathway and “Mito-Inflammation”: Recent studies have indicated a relationship between mitochondrial pathology and neuroinflammation and have shown that defective MQC and mtDAMPs contribute to neurodegeneration by activating the innate immune response and mitochondria-induced inflammation (“mito-inflammation”) [180]. These mtDAMPs activate cellular pattern recognition receptors (PRRs), including cGAS, to produce cGAMP, which then directly activates cyclic GMP-AMP synthase (cGAS)/stimulator of IFN genes (STING) signaling [181]. Among the mitochondrial DAMPs, considered danger signals, the proinflammatory molecules that induce and exacerbate the inflammatory response include: cardiolipin, cytochrome C, mtDNA, mitochondrial-derived reactive oxygen species (mtROS), and TFAM [181,182,183,184,185,186]. Activation of cGAS-STING leads to the induced transcription of IFN-stimulated genes and NF-kB-mediated inflammatory responses. STING is a cytoplasmic receptor that undergoes conformational changes and translocation from the ER to the endosomes and Golgi after binding to its ligand. The role of cGAS-STING is most extensively implicated in PD pathogenesis where defective mitochondrial clearance due to parkin or PINK1 loss-of-function mutations results in mtDNA leakage that activates the cGAS-STING pathway [187]. Furthermore, loss of STING prevents inflammation, motor defects, and neurodegeneration in both PINK1 KO and parkin KO mice [187]. Impaired lysosomal function may slow down the degradation of activated STING, a phenomenon which may further increase neuroinflammation in neurodegenerative diseases with coupled lysosomal and mitochondrial dysfunction. For example, mutations in the ER–lysosome lipid transport protein VPS13C cause early-onset PD and increased cGAS-STING pathway activation [188]. VPS13C knockout HeLa cells have been described as accumulating lysosomes with an altered lipid profile and defective STING degradation that, together with increased cytosolic mtDNA, can cause PD pathogenesis.

cGAS-independent pathways for the regulation of STING activity also relate to lipid metabolism. The availability of STING in the cytosol can be influenced by NPC1-dependent cholesterol transport. Before release into the cytosol, trafficking STING from the ER to the Golgi is dependent on its interaction with SREBP and SCAP [189]. Reduced cholesterol transport in NPC upregulates transport of the SREBP-SCAP complex to the Golgi, thus priming STING for release into the cytosol. As a more direct mechanism of STING regulation, excess STING is recruited to the lysosomes by NPC1 for degradation. Co-immunoprecipitation has highlighted a direct interaction between NPC1 and STING, and this interaction is maintained even during NPC1 truncation. NPC1 has 13 transmembrane proteins that can be grouped into three bundles that, when expressed individually, have been shown to maintain STING recruitment and degradation. A combination of these and cGAS-dependent pathways likely contributes to increased STING signaling in NPC1 knockout mice, worsening their neuropathology phenotype [189]. On the other hand, in NPC disease patients with mutations that do not prevent the trafficking of dysfunctional NPC1 to the lysosomal membrane, it remains to be seen whether the STING pathway could still be partially regulated through the degradation of STING by the lysosome in these patients.

Mitochondrial-Derived Vesicles: MDVs are a vesicular transport mechanism for inter-organelle communication. MDVs are cargo-selective vesicles which are 70–150 nm in diameter and bud off from mitochondria in a mechanism distinct from DRP1-mediated fission [190]. MDVs are formed in response to mitochondrial stress, including excessive ROS production, carbon monoxide toxicity, and response to lipopolysaccharide [191]. Snx9/OPA1-mediated MDVs contain IMM/matrix cargo and can be released as extracellular vesicles to trigger an IL-6 pro-inflammatory response [192]. However, upon antimycin A treatment to stimulate mitochondrial ROS production, the incorporation of mtDAMPs into MDVs and their subsequent secretion is selectively reduced to prevent a pro-inflammatory response [192]. Instead, the oxidized protein cargo is targeted by LE/lysosomes in a PINK1/parkin-dependent manner. While not being essential to all MDVs targeted by LE/lysosomes, PINK1 and parkin play a role in MDV formation and lysosomal targeting, a process that requires a complex of syntaxin17, Rab7, and VPS39/VPS41 subunits of the HOPS complex [129,193]. The cargo of PINK1/parkin MDVs is incorporated in a PINK1/parkin-dependent manner and consists of oxidized proteins following oxidative stress responses, which are specific to the type of damage. For example, VDAC is incorporated into MDVs resulting from cytosolic ROS, as opposed to complex III forming the cargo in response to antimycin A treatment [194]. MDVs are degraded at the lysosomes following their targeting by parkin, and this mechanism is independent of PINK1/parkin-mediated mitophagy and occurs in a much shorter timeframe (1–4 h) compared to mitophagy [129]. MDV transport is inhibited in models of PD, where PINK1 or parkin function is lost [129]. Given that the PINK1/parkin-mediated MDV formation and targeting to the lysosome for degradation is diminished in models of PD, and that neuroinflammation and increased IL-6 levels have been identified in PD patient tissue [195], it is possible that MDVs containing increased levels of oxidized IMM/matrix proteins may be secreted and play a role in the increased inflammatory phenotype and, therefore, the pathogenesis of PD.

## 7. Conclusions and Future Perspectives

Cells have evolved complex mechanisms for mitochondria:lysosome crosstalk, perhaps underpinning the importance of their coupled role in maintaining cellular homeostasis, with numerous examples where a defect in one organelle leads to dysfunction of the other in the context of disease. Recent advances have revealed a dense web of inter-related pathways involved in achieving coupling of mitochondrial and lysosome function and dysfunction. This requires complex coordination, the orchestration of which is yet to be fully elucidated.

While it is becoming increasingly apparent that nonvesicular communication at MLCs contributes significantly to mitochondria–lysosome crosstalk, we understand much less about how MLCs influence and are influenced by other crosstalk pathways, such as signaling or quality control through mitophagy and MDVs. Although it has been shown that mitophagy does not drive MLCs, the recently described roles of MLCs, for example in mitochondrial Ca^2+^ import, raise questions about the extent to which they may be involved in the regulation of mitophagy. Does buffering of cytosolic Ca^2+^ by MLC-mediated mitochondrial Ca^2+^ import restrict the induction of mitophagy? Or could extended MLCs act as a barrier to autophagosome formation, preventing recruitment of the autophagy machinery? Or, perhaps, Drp1 enrichment at MLCs may promote mitophagy.

The central role of mitochondrial ROS in inter-organelle crosstalk makes mitochondria-specific antioxidants an attractive therapeutic strategy for many of the diseases associated with coupled mitochondrial and lysosome dysfunction (summarized in Table 2). However, a 12-month clinical study targeting mitochondrial ROS for the treatment of PD was discouraging; the mitochondrial-targeted antioxidant MitoQ failed to slow disease progression [196]. Despite this, targeting mitochondrial function is rapidly gaining attention as a therapeutic approach (reviewed in [197]). Indeed, the mitochondrial-selective scavenger Mito-TEMP has been shown to ameliorate neuronal apoptosis induced by “PD toxins” that activate AMPK [198], and has been proposed as an approach for the prevention and treatment of PD [197,198].

Mitochondrial function is also thought to be the primary target of the recently FDA-approved NPC therapeutic N-acetyl l-leucine (Aqneursa), which, as well as correcting defective ATP production, concomitantly rescues lysosomal lipid storage phenotypes in NPC [199]. The exact mechanism of action of N-acetyl l-leucine remains unclear, but targeting the expanded MLCs identified in cellular models of NPC [5] could potentially contribute to the coupled restoration of mitochondria and lysosome function. As reviewed here and summarized in Table 2, dysregulation of MLCs has been strongly implicated in several diseases, yet we are not aware of any known diseases resulting directly from defects in MLC tethering proteins. In contrast, disease-causing mutations have been identified in genes encoding other MCS proteins including the ER tethering protein VAPB, mutations which are associated with a rare inherited form of the neurodegenerative disease amyotrophic lateral sclerosis (ALS) [200]. It is, therefore, possible that, as more MLC tethers are uncovered, disease-associated mutations in their genes will also be identified.

The dysregulation of MLCs in a growing number of diseases not only substantiates their key role in inter-organelle crosstalk but also raises the possibility of targeting specific MLC proteins as a novel therapeutic approach. Continued advances in the resolution of microscopes, coupled with the development of innovative new tools for the study of contact sites, will help unravel the intricate mechanisms of MLC regulation, paving the way for the development of new therapeutic strategies.

## Figures and Tables

**Table 1:** Interactions at mitochondria, ER, and lysosome membrane contact sites (MCSs).

**Mitochondria-Lysosome Contact Proteins**				
**Organelle Membrane Protein**	**Localization, Protein Function**	**Binding Partner at the Contact Site**	**MLC Function**	**Reference**
Rab7	LE/lysosomes, small GTPase, marks LE/lysosome PI3P, biogenesis, trafficking, positioning, and function regulator	DRP1—mitochondrial fission protein; TBC1D15—Rab7 GAP; Fis 1—recruitment of TBC1D15	Mitochondrial fission	[46,47]
Mitofusin-2 (MFN2)	Mitochondria, tethering, mitochondrial fusion	Unknown	Tethering, mitochondrial fusion	[48]
LAMP1	LE/lysosome, autophagy	GDAP—OMM glutathione S-transferase	Autophagy	[49,50]
STARD3 (MLN64)	LE/lysosome, ER to LE/lysosome cholesterol transport, and lysosome to mitochondria cholesterol import	Unknown	Contact has been implicated in cholesterol transport from lysosomes in NPC	[5,15]
VPS39	Vacuole, part of the vacuole and mitochondria patch (vCLAMP), fusion, and sorting	Tom40—channel-forming subunit of the translocase of the outer mitochondrial membrane (TOM) complex, protein sorting, it interacts with cholesterol–lipid complexes containing the StAR protein	Fusion and sorting	[51,52]
Vps13 (vacuole)	Vacuole, part of vCLAMP, lipid transport protein	MCP1—mitochondrial outer membrane protein that recruits Vps13 to the mitochondria	Lipid transport	[51]
**Mitochondria–ER Contact Proteins**				
**Organelle Membrane Protein**	**Localization, Protein Function**	**Binding Partner at the Contact Site**	**MERC Function**	**Reference**
IP3R	ER, Ca^2+^ release channel	VDAC1—mitochondrial metabolite channel; GRP75 (glucose-regulated protein 75)—component of the MQC and mitochondria-associated membrane (MAM); DJ-1—molecular chaperone, regulation of anti-oxidative stress reaction	Mitochondria to ER Ca^2+^ release	[44]
SERCA2	Ca^2+^-ATPase	MFN2—mitochondria, tethering, mitochondrial fusion	Tethering, mitochondrial fusion	[53]
PDZD8	Lipid transport protein	FKBP8—mitophagy receptor that recruits LC3A to damaged mitochondria	Mitophagy	[54,55]
VAP	ER tethering receptors through their FFAT motif	PTPIP51—mediating IP3R-mediated delivery of Ca^2+^ from the ER to the mitochondria, mitochondrial ATP production, and autophagy	Ca^2+^ transport, mitochondrial lipid transport protein, mitochondrial dynamics	[56]
**Lysosome–ER Contact Proteins**				
**Organelle Membrane Protein**	**Localization, Protein Function**	**Binding Partner at the Contact Site**	**MCS Function**	**Reference**
Annexin-A1	Ca^2+^-dependent phospholipid-binding protein, anti-inflammatory mediator	S100A11—Ca^2+^-binding protein	Ca^2+^ transport, facilitating PTP1B to moderate effects at the endosome	[57]
EGFR (epidermal growth factor receptor)	Plasma membrane (PM), endosomes, lysosome, receptor tyrosine kinase, growth factor receptor	PTP1B—tyrosine phosphatase, dephosphorylates EGFR, and ESCRT-0	Regulates endosome maturation and receptor tyrosine kinase signaling	[58]
NPC1	LE/lysosome, cholesterol egress	Gramd1b/ORP5	Sterol and phospholipid transport	[5]
ORP1L	LE/lysosome, oxysterol-binding protein family, it interacts with Rab7 and transports cholesterol, endosomal positioning in RAB7/RILP complex	VAP/MOSPD2—binding to FFAT motif-containing proteins for tethering	Cholesterol trafficking under low cholesterol conditions	[59,60]
Rab7	LE/lysosome, small GTPase, marks LE/lysosome PI3P, biogenesis, trafficking, positioning, and function regulator	Protrudin—binds to Rab7 and PI3P in the LELys membrane, it recruits kinesin-1 to LE/lysosome, it promotes anterograde transport of lysosomes to the PM in neurite outgrowth; PDZD8—lipid transport protein, it binds to VAP, it recruits kinesin-1	Lipid transport	[61,62]
STARD3 (MLN64)	LE/lysosome, mediates ER to LE/lysosome, cholesterol transport, and mitochondrial cholesterol import	VAP/MOSBP2—binding to FFAT motif-containing proteins for tethering	ER to LE/lysosome cholesterol transport	[59,63,64]

**Table 2:** Mitochondria–lysosome coupled dysfunction in disease.

Disease	Causal Factors	Evidence for Coupled Dysfunction	Model	Reference
**Niemann**–**Pick type C (NPC)**—lysosomal storage disorder	Loss of function mutations in lysosomal cholesterol transport proteins NPC1 (95% of patients) or NPC2 (5% of patients)	Increased mitochondrial cholesterol	CHO and NPC1-deficient 4-4-19 cell lines	[15]
		Altered mitochondrial morphology, reduced mitophagy	NPC1 and NPC2 patient-specific iPSCs	[16]
		Mitochondrial ROS levels increased, mitochondrial biogenesis reduced, reduced respiration	NPC patient fibroblasts, *Npc^−/−^* mice	[17]
		Reduced mitochondrial membrane potential, reduced ATP synthesis and ATP synthase activity	BALB/c NPC1 mouse model	[18]
		Increased physical contact between lysosomes and mitochondria	NPC1-inhibited HeLa, NPC patient fibroblasts	[5]
**Mucolipidosis type IV**—lysosomal storage disorder	Transient receptor potential mucolipin 1 (TRPML1) lysosomal Ca^2+^ efflux channel	Increased physical contact between lysosomes and mitochondria, reduced mitochondrial Ca^2+^ uptake	MLIV patient fibroblasts	[42]
		Mitochondrial fragmentation, reduced mitochondrial membrane potential, reduced respiration, increased expression of MICU1	*TRPML1^−/−^* NK cells	[70]
		Mitochondrial fragmentation, increased ROS production, lipid peroxidation	siRNA-induced TRPML1 knockdown in RPE1 cells	[123]
**Age-related macular degeneration**—degenerative blinding disease	Multi-factorial (age and complex environmental and genetic risks)	Increased ROS production by mitochondria increasing lipofuscin aggregations and limited degradation by lysosomes, limiting autophagy	Multiple	Reviewed in: [124,125]
	Complement factor H (Y402H-CFH)	Mutation causes impaired lysosome maturation and lowered cathepsin D activity, coupled with reduced respiration	iPSC RPE	[126]
**Parkinson’s disease**—neurodegenerative disease	Lysosomal glucocerebrosidase GBA1	Increased physical contact between mitochondria and lysosomes, elevated lysosomal pH, reduced respiration	GBA1-PD patient neurons	[30]
		Reduced mitochondrial Ca^2+^ uptake due to reduced MCU1 expression, increased ROS production	*Gba1^−/−^* neurons	[14]
		Altered mitochondrial morphology, reduced mitochondrial membrane potential	GBA1-inhibited SHSY-5Y	[127]
	PINK1 (mitochondrial)	Mitophagy impairment	*PINK1^−/−^* MEF	[128]
		Increased LAMP1-positive vacuoles	*PINK1^−/−^* MEF	[19]
		Reduced MDV formation and altered kinetics	siRNA-induced PINK1 knockdown in HeLa	[129]
**Charcot**–**Marie**–**Tooth type 2B disease**— axonal sensorimotor neuropathy	Rab7 GTPase (V162M mutant)	Elongated mitochondria–lysosome contact sites, enlarged lysosomes, reduced mitochondrial motility, altered mitochondrial morphology	Rab7 V162M peripheral sensory neurons, Rab7 V162M knock-in mice	[130]
	Ganglioside-induced differentiation-associated protein 1 (GDAP1) mitochondrial	Impaired autophagy, enlarged lysosomes	SHSY-5Y, embryonic motor neurons from *gdap1^−/−^* mice	[49]
		Altered mitochondria–lysosome contact sites: increased in the T157P mutant and decreased in the R161H mutant	HEK293T cell overexpression of mutant GDAP1	[50]
